# Social Priming Improves Cognitive Control in Elderly Adults—Evidence from the Simon Task

**DOI:** 10.1371/journal.pone.0117151

**Published:** 2015-01-30

**Authors:** Daniela Aisenberg, Noga Cohen, Hadas Pick, Iris Tressman, Michal Rappaport, Tal Shenberg, Avishai Henik

**Affiliations:** Department of Psychology, and the Zlotowski Center for Neuroscience, Ben-Gurion University of the Negev, Beer Sheva, Israel; University College London, UNITED KINGDOM

## Abstract

We examined whether social priming of cognitive states affects the inhibitory process in elderly adults, as aging is related to deficits in inhibitory control. Forty-eight elderly adults and 45 young adults were assigned to three groups and performed a cognitive control task (Simon task), which was followed by 3 different manipulations of social priming (i.e., thinking about an 82 year-old person): 1) negative—characterized by poor cognitive abilities, 2) neutral—characterized by acts irrelevant to cognitive abilities, and 3) positive—excellent cognitive abilities. After the manipulation, the Simon task was performed again. Results showed improvement in cognitive control effects in seniors after the positive manipulation, indicated by a significant decrease in the magnitude of the Simon and interference effects, but not after the neutral and negative manipulations. Furthermore, a healthy pattern of sequential effect (Gratton) that was absent before the manipulation in all 3 groups appeared after the positive manipulation. Namely, the Simon effect was only present after congruent but not after incongruent trials for the positive manipulation group. No influence of manipulations was found in young adults. These meaningful results were replicated in a second experiment and suggest a decrease in conflict interference resulting from positive cognitive state priming. Our study provides evidence that an implicit social concept of a positive cognitive condition in old age can affect the control process of the elderly and improve cognitive abilities.

## Introduction

Imagine an 82 year-old man enjoying his retirement. He attends online bridge competitions, reads interesting books, and solves crossword puzzles. His memory is as good as it was when he was 30, and he knows it. What would you think of such a person?

More importantly, do you think that thinking about him has an influence on *you*? And, if there is such an influence, could it be helpful to you and improve your performance? The present study aims to examine the influence of thinking about another person (i.e., a social concept) on cognitive performance in old age.

### Cognitive Control and Aging

Cognitive control, a component of executive functions, has been found to be affected by aging [1–3]. Cognitive control is related to conflict monitoring—the ability to suppress irrelevant information [4] and engage selective attention. Commonly, we orchestrate among conflicting sources of information or demands. For example, we may have to decide which object, dimension of an object or event needs to be attended to, and which should be ignored, inhibited, or deferred for later processing. These situations require cognitive control, particularly inhibition. Hasher and Zacks [2] have suggested that age-related cognitive impairments result from the weakening of a general inhibitory system. This notion has been supported by other studies as well [5–6]. Recently, Maylor, Birak, and Schlaghecken [7] found evidence that age- related deficits in inhibitory control generalize to conflicts arising at different levels of perceptual and motor processing.

### Simon Task

One of the simplest tasks employed in studies of control and inhibition is the Simon task [8]. In a visual Simon task, participants are presented with a colored stimulus that appears on the left or the right of fixation, and are required to press a left or right key to report its color [9–11]. This arrangement creates two trial types: 1) congruent trials, when the stimulus is presented on the side of the required key-press; and 2) incongruent trials, when the stimulus is presented on the side opposite of the required key-press. Participants respond faster to congruent than incongruent stimuli, a reaction time (RT) difference called the Simon effect. Adding a neutral stimulus (third trial type, positioned at the central vertical meridian of the screen) allows for examining components of the Simon effect. Since a neutral stimulus should not present a conflict [12], we expect that performance in response to it should be better than for conflict trials, and worse than for facilitated trials. This set-up allows for distinguishing between components of facilitation (neutral vs. congruent) and interference (incongruent vs. neutral) that are present in the Simon effect, and for testing the magnitude of each component. A neutral condition was introduced as a mean of manipulating control [13]. The authors suggested that proportionally more neutral trials would induce a lenient attitude in participants, and reduce control. In line with this suggestion, they found that increasing the number of neutral trials increased interference magnitude, suggesting a reduction of control.

The most common explanation for the Simon effect is related to inhibitory control processes, as a means of solving the spatial conflict created [14–15]. Accordingly, one would expect changes in the Simon effect with aging. Indeed, previous results suggest that aging negatively impacts Simon task performance. Van der Lubbe and Verleger [3] found a larger Simon effect in older individuals, which could not be accounted for by generalized psycho-motor slowing due to aging. Bialystok, Craik, Klein, and Viswanathan [16] found that monolingual elderly adults showed an increased Simon effect compared to bilingual adults and suggested that aging effects on an inhibitory process can be modified by lifelong habits and practice with inhibition of interference from competing languages. Aisenberg, Sapir, d’Avossa and Henik [17] compared young and elderly adults in the Simon task using a neutral condition and found a greater Simon effect, particularly due to a greater interference component, in elderly adults.

### Effects of Social Priming on Automatic Cognitive Processes

There is evidence that priming a social concept triggers automatic behavior that is consonant with the primed concept [18]. For example, after Bargh, Chen, and Burrows [19] primed participants with the social concept “elderly,” participants behaved like elderly people and walked at a slower pace for 5 minutes. Dijksterhuis and van Knippenberg [20] found that when participants were primed with the social concept “professor”, they performed better in a general knowledge task. Also, when participants were primed with the social concept of “soccer hooligans” who are perceived as stupid, a decrease in performance in a general knowledge task was viewed. These changes lasted up to 15 minutes. The replication of these studies is mixed, showing failure [21–22] and success, described by Stroebe, and Strack [23] as successful conceptual replications of the study of Bargh et al [19] [24–28], and the study of Dijksterhuis and van Knippenberg [20] [29–34].

Levy [35] primed elderly individuals with age stereotypes (e.g., wisdom, decrepit). She found that participants primed with negative age stereotypes performed worse than those primed with positive age stereotypes in memory tasks. Recently, Goldfarb, Aisenberg, and Henik [36] conducted a Stroop task, priming participants with a social priming concept of dyslexia. In their study, participants were asked to perform a Stroop task (they were presented with colored words and requested to respond to the printed colors while ignoring the meaning of the written words), followed by a manipulation of a dyslectic person, a painter, or a writer, and then they performed another session of the Stroop task. It was found that the known Stroop effect (RT difference between incongruent conflicting word and color trials and congruent matching word and color trials), created because reading is an automatic process, was eliminated for participants primed with dyslexia, in the first block after manipulation. This study was fully replicated recently [37]. This strongly suggests that social priming can affect automatic processing, particularly inhibition.

### The Present Study

In line with the notion that social priming has an effect on automatic cognitive processes, the present study examined whether social priming could affect the cognitive process of inhibition in healthy elderly adults. Levy [38] suggested that aging stereotypes are beliefs about elderly people as a category, that can operate “below awareness” (p. 203) and become self-stereotypes in old age. In a study by Levy, Slade, Murphy, and Gill [39], elderly adults with positive age stereotypes were44% more likely to fully recover from severe disability than those with negative age stereotypes. We used a Simon task under conditions of social priming. As a social priming manipulation we used the concept of an elderly adult in a negative, neutral, or positive cognitive state. Our hypotheses are specific to the positive manipulation influence but we employed neutral and negative manipulations as well as controls. If a social concept influences inhibitory control, we would expect a decrease of conflict interference after a positive manipulation, compared to neutral and negative manipulations, resulting in a smaller congruency (Simon) effect. In order to better understand the impact of social priming on inhibitory control we shall, by using a neutral condition, address the effects in terms of facilitation and interference.

Sequential dependencies are another measure of cognitive control. Larger congruency effects are found in trials following congruent than incongruent trials, suggesting that cognitive control is engaged not only at the time of stimulus presentation, but also affects preparatory processes that follow it [40–41]. Congruency effects following neutral trials were found to resemble those after congruent trials in the Simon task [42–43], though Lamers and Roelofs [44] showed them to resemble the after-incongruent effects in the Stroop and flanker tasks, challenging the view of Botvinick et al. [40].

In the above-mentioned studies, young adults showed a significant congruency effect after congruent trials that decreased to a non-significant effect after incongruent trials. This effect is also called the adaptation effect (or Gratton effect), as it reflects improved performance from one conflict trial to the following conflict trial. Aisenberg et al. [17] found impaired sequential dependencies in elderly adults, as they showed a significant Simon effect both after congruent and incongruent trials. This was taken as an indication of a relatively less efficient inhibitory process.

## Experiment 1

In the context of the present study, if social priming affects cognitive control, we would expect a smaller Simon effect and interference following the implementation of the positive manipulation compared to the negative manipulation. Additionally, we would expect a normal pattern of sequential dependencies after receiving the positive manipulation, as elderly adults will show no Simon effect after incongruent trials, but will show it after congruent trials.

At the beginning of the experiment, participants performed a two-session Simon task. During the break between the two sessions, they were asked to fill out a “social psychology” questionnaire supposedly related to another study. Three social priming manipulations (negative, neutral, or positive) were used, asking participants to write, within a five-minute period, their thoughts regarding the following: [Negative] *“Joseph, 82 years old, senses a deterioration of his abilities. His memory is impaired and his thinking is not as fast as it used to be. Joseph is frustrated about this, and about the fact that he can no longer solve crossword puzzles as he always loved.”*

[Neutral] *“Joseph, 82 years old, is going to his routine examination in a health maintenance organization (HMO). He lives across the street from the HMO, and before an examination he wakes up early and packs an apple and a bottle of water in his bag.”*

[Positive] *“Joseph, 82 years old, is enjoying his retirement. Every once in a while he meets friends who tell him they don’t feel as well as they used to feel, and that their memory is impaired. Joseph doesn’t share this sense at all. His memory is as good as ever, he easily solves cross-words puzzles, and he enjoys thinking games.”*

“For the next five minutes, think and write about Joseph’s everyday life. Describe his daily routine. What does he do well, and what does he do poorly?”

Each manipulation was given to 10 different naïve seniors who rated its’ valence on a 10 positive-negative scale (1 = most negative, 10 = most positive). The Negative manipulation scored 1.7 (SD = 0.8), the neutral manipulation scored 4.9 (SD = 0.5) and the positive manipulation scored 10 (SD = 0).

It is important to distinguish effects of cognitive social priming from effects of motivation and mood. Social priming of motivation was found to have a positive effect on executive control (see [45]; word puzzles consisting of motivational words improved performance in the Wisconsin Cards Sorting Test—WSCT). Additionally, effects of positive and negative mood were found to influence executive function performance. However, the results of mood studies were inconsistent [46–48]. To insure that our priming manipulation did not affect motivation or mood, we conducted a manipulation check in which elderly adults filled mood and motivation questionnaires before and after responding to the five-minute neutral or positive manipulation (see S1 File). No motivation or mood-induced differences were found.

## Materials and Methods

This study was conducted under a protocol approved by the Soroka Helsinki Ethics Committee. All participants have given a written informed consent prior to their participation.

### Participants

51 healthy elderly adults were initially recruited, leading to a final sample of 48 (*mean age* = 71.9 years, *SD* = 5.8) and a control group of 45 young adults (*mean age* = 23.8 years, *SD* = 2.7), 35 of them grandchildren of the elderly adults were randomly assigned to three groups and participated in the experiment for payment. The choice to have 16 elderly adults in each group follows the amount of participants in the study of Goldfarb et al. [36]. All had normal or corrected-to-normal vision, without color blindness. The elderly adults groups did not differ in their level of education and socio-economic status (the participants with different levels were recruited separately and randomly assigned to all three groups). All were naïve regarding the purpose of the experiment.

### Stimuli

**Fig 1:**
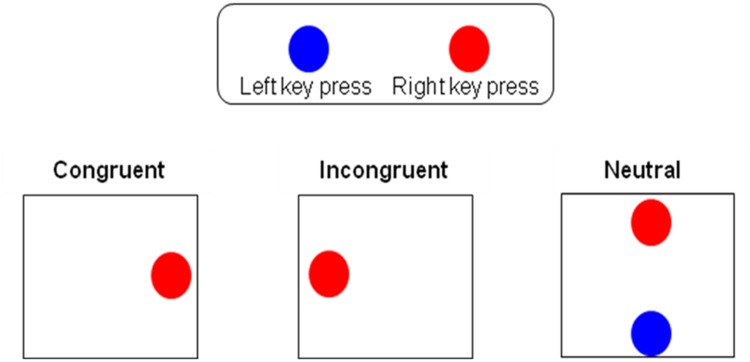
Three levels of congruency in the Simon task: a) Congruent b) Incongruent c) Neutral.

Each stimulus was a red or blue patch, 5° in diameter, displayed at one of four locations 13° from the center of the screen: left or right central horizontal meridian of the screen or top or bottom central vertical meridian of the screen (see Fig. 1). Consequently, there were 2 different incongruent stimuli (when the patch appeared on the side opposite of the required key-press), 2 different congruent stimuli (when the patch appeared on the side corresponding to the required key-press) and 2 different neutral stimuli (when the patch appeared on vertical position, irrelevant to a horizontal response). A block of 91 trials was created pseudo-randomly, containing at least 30 trials of each condition (congruent, incongruent and neutral), to allow generating the sequence of trials so that each of the nine possible sequential pairings between trial types was present at least 10 times (previous trial congruent and current trial congruent; previous trial congruent and current trial neutral and so on). A practice block consisting of 16 trials preceded experimental blocks.

### Procedure

Data collection and stimulus presentation were controlled by a Compaq computer with an Intel Pentium III central processor. Stimuli were presented on a Compaq S510 monitor. A keyboard was placed on a table between the participant and the monitor. Colored stickers were placed on the keyboard keys according to the colors they represented, in a balanced layout. For half of the participants the “D” key represented red and the “L” key represented blue, and for the other half of the participants the “D” key represented blue and the “L” key represented red. The participants pressed the “D” key with their left index finger and the “L” key with their right index finger.

**Fig 2:**
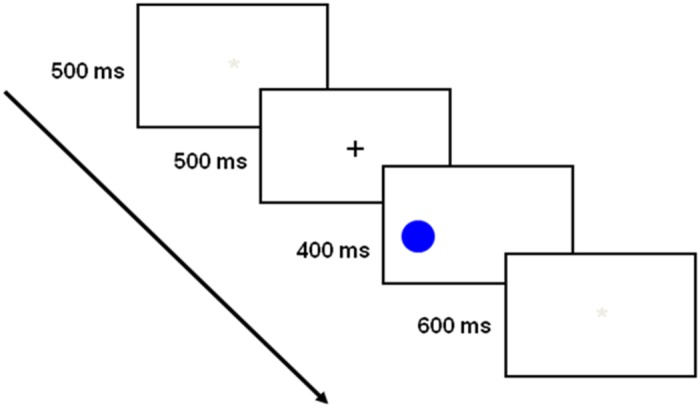
Trial procedure. The presented target represents a congruent condition.

At the beginning of the experiment, participants performed the Ishihara Color Blindness Test to examine color vision and elderly adults were screened with the Mini-Mental Status Exam [49] to rule out cognitive deficits. We decided that participants who score less than 27 were will be excluded. 3 participants were excluded, and the remaining 48 participants were then instructed to perform a Simon task that was composed of two sessions, each containing two separate blocks of 91 trials. During the break, they were asked to fill out a questionnaire supposedly related to another study. At the beginning of the Simon experiment, the participants were instructed to press the key on the keyboard that matched the stimulus color on screen. The participants were asked to respond as quickly as possible without making mistakes. Participants were run individually. They sat approximately 60 cm from the computer screen. Before the beginning of the experimental blocks, the participants practiced on 16 Simon trials. Each trial started with a blank screen for 500 ms, followed by a 500 ms presentation of a fixation black cross at the center of the screen. After the fixation point disappeared, the stimulus appeared either to the right, left, above or below the central position of the screen for 400 ms. The target stimulus was followed by a 600 ms blank screen during which the participant had to respond (see Fig. 2). On incorrect trials, in the practice block only, a 1,000 ms feedback message with the word “error” appeared before the next trial began. RT in milliseconds was measured by the computer from stimulus onset until the participant’s response.

After the performance of the Simon task, participants were asked to perform a “social psychology” questionnaire task, supposedly related to another study. In this phase we applied the social priming technique as used in the study of Goldfarb et al. (2011). They were randomly assigned to each of three social priming manipulations (negative, neutral or positive) and were asked to write, within a five-minute period, their thoughts regarding the everyday life of the person described. Each manipulation group consisted of 16 elderly adults and 15 young adults.

At the end of this task, which was timed by the experimenter with a stop watch, participants were asked to perform another session of the Simon task. This session was identical to the first one described above, excluding practice trials.

## Results

Mean RTs for correct responses as well as two standard deviations above and below mean RT were calculated for each participant in each condition. Based on these calculations, results below 100 ms or above two standard deviations were excluded from the analysis. Analyzing two standard deviations below mean results (in all participants) in RTs that are below 100ms. Since we know that 100 ms RTs are too fast responses we drew the line there. The overall error rate was low (0.06), and errors were not analyzed.

Young adults were overall faster than elderly adults, *F*(1, 87) = 73.01, *η* ^2^_p_ = .45, *MSE* = 125,779, *p* = .0001. A main effect of congruency was found in both young and elderly adults, *F*(2, 84) = 78.9, *η* ^2^_p_ = .65, *MSE* = 574, *p* = .0001, and *F*(2, 90) = 116.76, *η* ^2^_p_ = .72, *MSE* = 1,451, *p* = .0001, respectively, as congruent trials were overall faster (young: 376 ms, elderly: 509 ms) than neutral trials (young: 388 ms, elderly: 545 ms), *F*(1, 42) = 36.52, *MSE* = 593.7, *p* = .0001, and *F*(1, 45) = 72.95, *MSE* = 1,664.4, *p* = .0001, respectively, and neutral trials were faster than incongruent trials (young: 402, elderly: 568 ms), *F*(1, 42) = 66.8, *MSE* = 353.5, *p* = .0001, and *F*(1, 45) = 66.57, *MSE* = 790.9, *p* = .0001, respectively. The interaction of age and congruency was also significant, *F*(1,87) = 22.43, *η* ^2^_p_ = .20, *MSE* = 1,470, *p* = .0001, replicating the finding that the Simon effect was larger for elderly compared to young adults, *F*(1,87) = 31.97, *MSE* = 2,050.81, *p* = .0001 [3]. Specifically, the interference component was significantly larger for elderly compared to young adults, *F*(1,87) = 31.97, *MSE* = 2,050.81, *p* = .0001, replicating the results of Aisenberg et al. [17].

**Fig 3:**
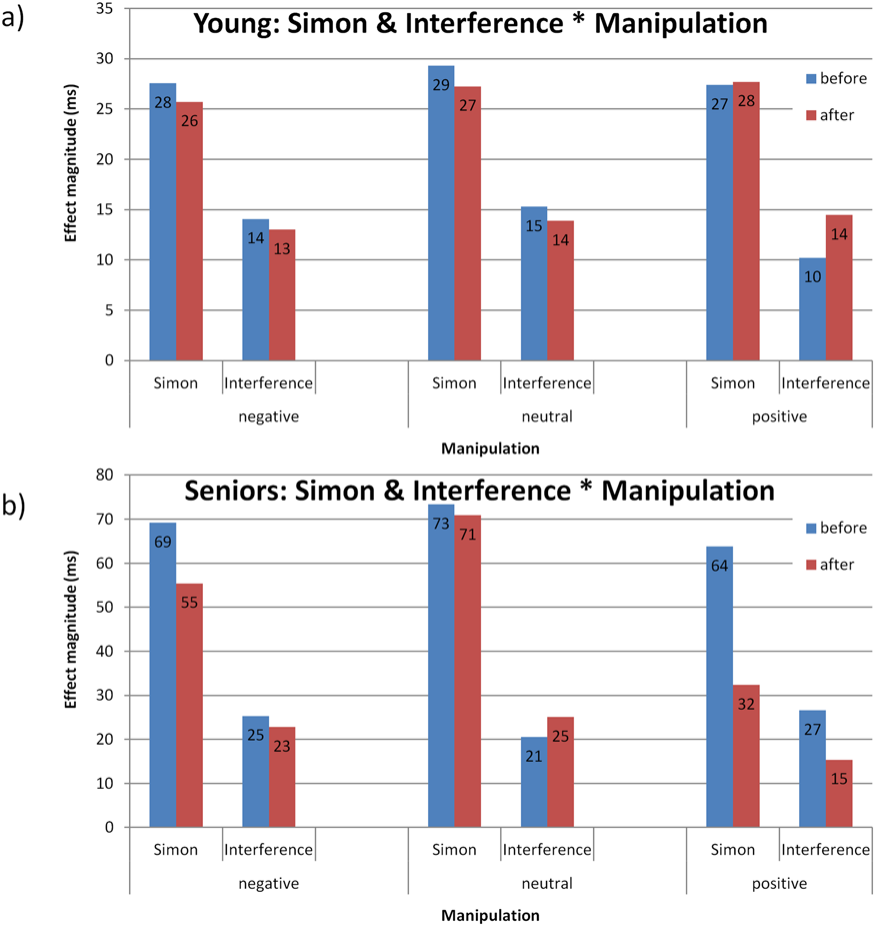
Experiment 1: Simon and interference effects magnitude as a function of manipulation and session in a) young adults and b) elderly adults. In the elderly adults group a significant reduction in the Simon effect (incongruent RTs—congruent RTs) and interference (incongruent RTs—neutral RTs) was found only after the positive manipulation and not after negative or neutral manipulation.

**Fig 4:**
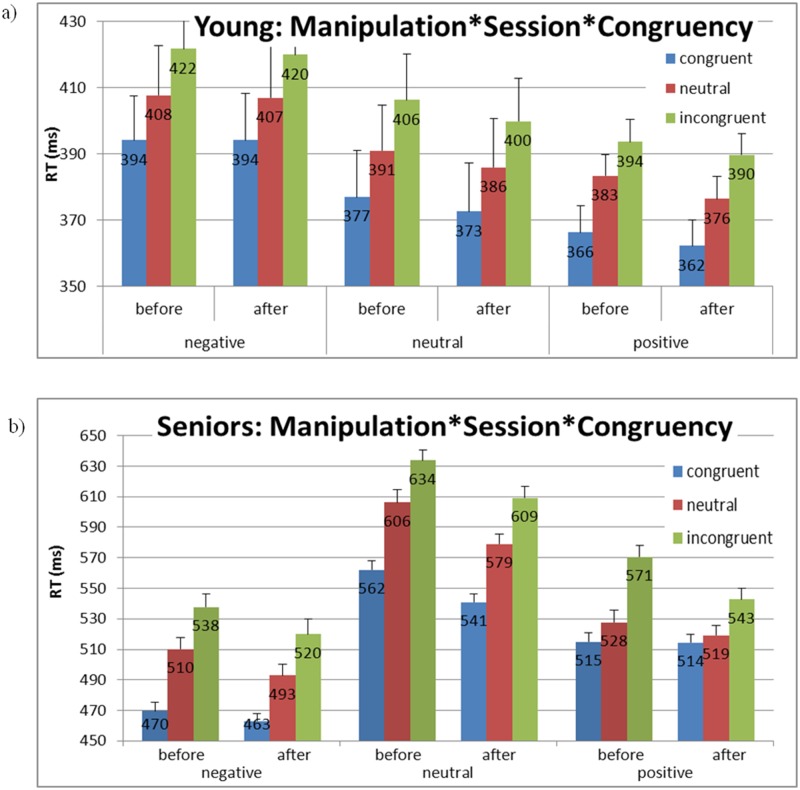
Experiment 1: Mean RTs and standard error (SE) for congruency as a function of manipulation and session for a) young adults and b) elderly adults (detailed data extending Fig. 3). In the elderly adults group only the Simon effect (incongruent RTs—congruent RTs) differed significantly between positive manipulation and the neutral and negative manipulations. More importantly, the interference effect (incongruent RTs—neutral RTs) differed significantly between the positive manipulation and the neutral and negative manipulations. To insure that the significant decrease after the positive manipulation was not a result of baseline differences, we compared interference effects.

The four-way interaction between age, manipulation, session, and congruency was significant, *F* (4,174) = 2.61, *η* ^2^_p_ = .056, *MSE* = 555, *p* = .036. For simplicity, age groups were further analyzed separately. An analysis of variance (ANOVA) was carried out on mean RTs for congruency (congruent, neutral, incongruent) X session (before manipulation, after manipulation) X manipulation (negative, neutral, positive). In the elderly adults group the three-way interaction between session, manipulation and congruency was significant, *F*(4, 90) = 2.291, *η* ^2^_p_ = .09, *MSE* = 536, *p* = .008 (see Fig. 3b and Fig. 4b). As we expected, the Simon effect (incongruent RTs—congruent RTs) differed significantly between the positive manipulation and the neutral and negative manipulations, *F*(1, 87) = 6.12, *MSE* = 421.98, *p* = .015. More importantly, the interference effect (incongruent RTs—neutral RTs) differed significantly between the positive manipulation and the neutral and negative manipulations, *F*(1, 87) = 5.82, *MSE* = 445.59, *p* = .017 (see distribution plots for every participant in each manipulation in S1 Fig. for seniors and S2 Fig. for young adults). No effect of manipulation was found in the young adults group, *F*(4, 84) < 1 (see Fig. 3a and Fig. 4a).

Within the positive manipulation group the effects of congruency were tested between sessions: the Simon effect was smaller after the positive manipulation than before it (see Fig. 3), *F*(1, 87) = 13.93, *MSE* = 421.98, *p* = .0003. More importantly, a significant difference was found for the interference effect between sessions, *F*(1, 87) = 6.39, *MSE* = 445.59, *p* = .013, in which the interference effect was smaller after, as compared to before, the positive manipulation. These effects were not found in the negative and neutral manipulations (Simon and interference effects in negative and neutral manipulations, respectively: *F*(1,87) < 1, *F*(1,87) < 1; *F*(1,87) < 1, *F*(1,87) < 1).

**Fig 5:**
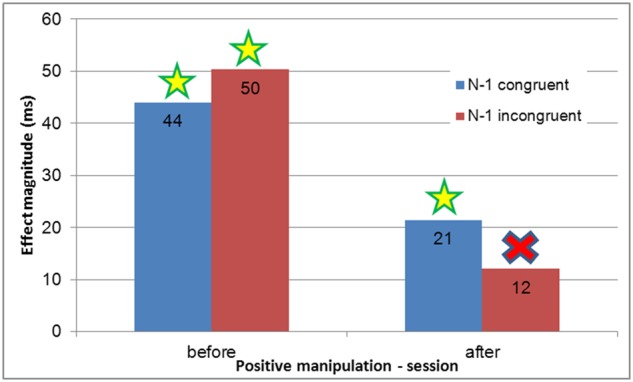
Experiment 1: Simon effect magnitude as a function of previous congruency between sessions in the positive manipulation for seniors. Before the manipulation, the Simon effect was significant after both congruent and incongruent trials (maladaptive pattern of sequential analysis). After the manipulation, the Simon effect was significant after congruent, but not after incongruent, trials (appearance of an adaptive pattern).

**Fig 6:**
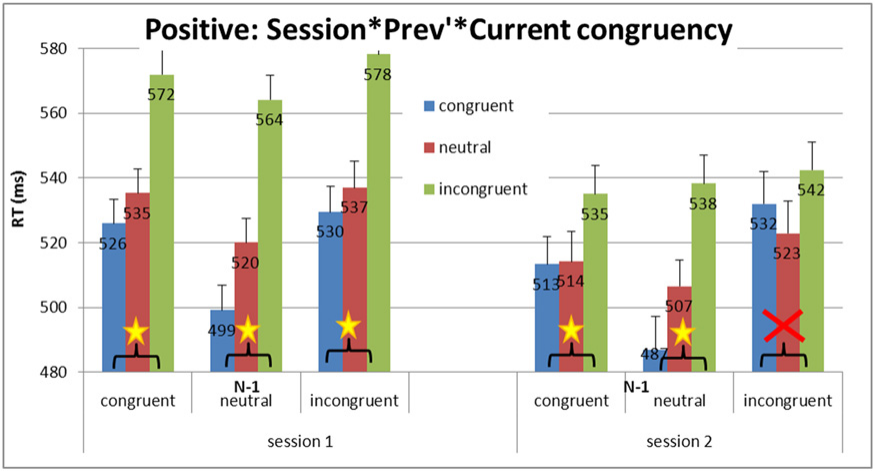
Experiment 1: Mean RTs and SE for congruency as a function of previous congruency between sessions in the positive manipulation (detailed data extending Fig. 5). The maladaptive pattern of sequential analysis was present before the manipulation (Simon effect was significant after both congruent and incongruent trials), while an adaptive pattern of sequential analysis was present after the positive manipulation (Simon effect was significant after congruent, but not after incongruent, trials.

To measure whether the positive manipulation affected the inhibitory process between trials, sequential analysis was carried out for the positive manipulation group (see Fig. 5 and Fig. 6). Planned comparisons of the Simon effect and interference effect were carried out between the ‘before manipulation’ session and the ‘after manipulation’ session. While young adults showed an unchanged Gratton pattern of sequential analyses in all sessions and manipulations, *F*(1,42) < 1 (Simon effect significant after congruent but not after incongruent trials), a maladaptive Gratton was observed for elderly adults before the manipulation (the Simon effect was significant after both congruent and incongruent trials, *F*(1, 15) = 5.67, *MSE* = 2730.66, *p* = .03, and *F*(1, 15) = 11.62, *MSE* = 1743.85, *p* = .003, respectively, replicating the results of Aisenberg et al. [17]. Nevertheless, in line with our hypothesis, an adaptive pattern of sequential analysis was observed after the positive manipulation (Simon effect was significant only after congruent but not after incongruent trials, *F*(1, 15) = 5.61, *MSE* = 654, *p* = .03, and *F*(1, 15) < 1, respectively. It is worth mentioning here that the Simon effect was also significant after neutral trials, both before and after the manipulation, *F*(1, 15) = 111.58, *MSE* = 379.58, *p* = .0001, and *F*(1, 15) = 22.78, *MSE* = 909.88, *p* = .0002, respectively, resembling the congruent condition, and thus replicating the findings of Aisenberg and Henik [42], and strengthening those of Botvinick et al. [40].

One last result worth pointing out is related to how long the manipulation effect lasts. In the study of Goldfarb et al [36] the decrease in the Stroop effect lasted only for the first block of the second session, after the manipulation. Here this was not the case. We conducted another analysis testing differences between the two blocks in the second session after the positive manipulation. Our results did not yield a significant difference between the blocks, suggesting a longer lasting effect of the positive manipulation in old age (decreased interference in both blocks of the second session *F*(1, 45) < 1, and a similar Gratton pattern *F*(1, 45) < 1). This would be further discussed in the discussion.

The results of this experiment are striking. As the field of social priming is by itself under debate [50] a need for strong support is in order. To allow better confidence in our suggestion that priming a positive cognitive state provides improvement of cognitive control performance in elderly we attempted to replicate our results.

## Experiment 2

Experiment 2 was conducted in attempt to replicate our striking results. In particular we expected that the positive manipulation would produce a change in the interference as well as the Gratton effects in the elderly, suggesting specific improvement of control measurements after positive cognitive priming.

## Materials and Methods

### Participants

36 healthy elderly adults who did not take part in experiment 1 were randomly assigned to two groups (positive and neutral, *mean age* = 72 years, *SD* = 6.8 and *mean age* = 73 years, *SD* = 6.2, respectively) and participated in the experiment for payment. This time, the choice to have 18 elderly adults in each group followed a statistical power analysis using G*Power [51] to calculate the appropriate N. The type of power analysis was a priori, announcing an expected effect size f of 0.1 for the four-way interaction between manipulation, session, previous congruency and current congruency, considering α error probability = .05 and a satisfactory power 1-β error probability = 0.9, in 2 manipulation groups (neutral and positive).

All participants had normal or corrected-to-normal vision, without color blindness, and scored 27/30 or above in the MMSE. The groups did not differ in their level of education and socio-economic status. All were naïve regarding the purpose of the experiment.

### Stimuli and Procedure

Stimuli and procedure were identical to those of Experiment 1 repeating the neutral and positive manipulation.

## Results

Mean RTs for correct responses as well as two standard deviations above and below mean RT were calculated for each participant in each condition. Based on these calculations, results below 100 ms or above two standard deviations were excluded from the analysis. The overall error rate was low (0.05), and errors were not analyzed. Overall, the results replicated those of Experiment 1.

A main effect of congruency was found *F*(2, 68) = 180, *η* ^2^_p_ = .71, *MSE* = 2300, *p* = .0001, as congruent trials were overall faster (568 ms) than neutral trials (580 ms), *F*(1, 34) = 36, *MSE* = 410, *p* = .0001, and neutral trials were faster than incongruent trials (607 ms), *F*(1, 34) = 22, *MSE* = 382.4, *p* = .0001.

**Fig 7:**
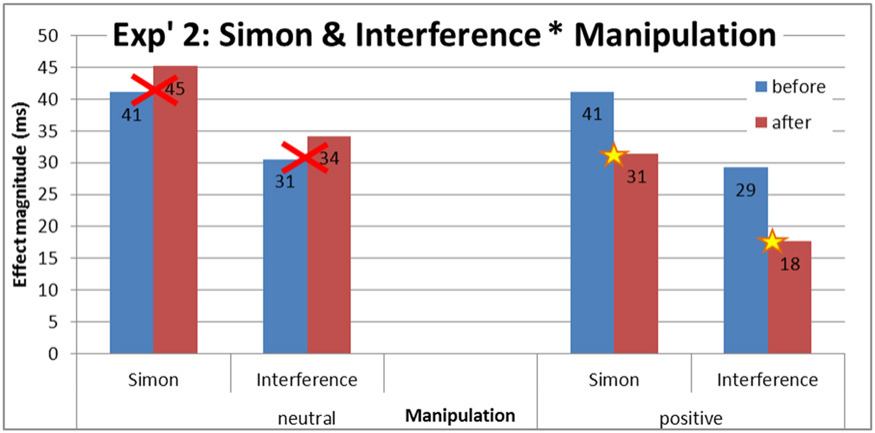
Experiment 2: Mean RTs and standard error (SE) for congruency as a function of manipulation and session. The Simon effect (incongruent RTs—congruent RTs) differed significantly between positive manipulation and the neutral manipulation. More importantly, the interference effect (incongruent RTs—neutral RTs) differed significantly between the positive manipulation and the neutral manipulation.

**Fig 8:**
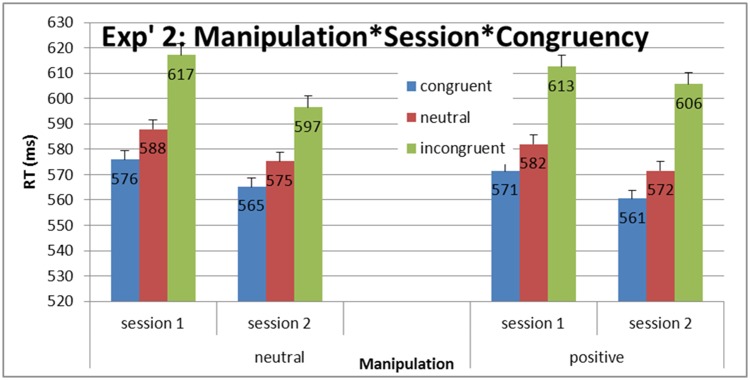
Experiment 2: Simon and interference effects magnitude as a function of manipulation and session. A significant reduction in the Simon effect (incongruent RTs—congruent RTs) and interference (incongruent RTs—neutral RTs) was found only after the positive manipulation and not after the neutral manipulation.

An analysis of variance (ANOVA) on mean RTs for congruency (congruent, neutral, incongruent) X session (before manipulation, after manipulation) X manipulation (neutral, positive) was found to be significant, *F* (2,68) = 5.44, *η* ^2^_p_ = .13, *MSE* = 177, *p* = .006 (see Fig. 7). The difference in Simon effect (incongruent RTs—congruent RTs) between the positive manipulation and the neutral manipulation was marginally significant, *F*(1, 34) = 1.2, *MSE* = 285.65, *p* = .059. More importantly, the interference effect (incongruent RTs—neutral RTs) differed significantly between the positive manipulation and the neutral manipulation, *F*(1, 34) = 6.4, *MSE* = 382.4, *p* = .016 (see Fig. 8; for distribution plots for every participant in each manipulation see S3 Fig.).

Within the positive manipulation group the effects of congruency were tested between sessions: the Simon effect was smaller after the positive manipulation than before it (see Fig. 8), *F*(1, 34) = 4.46, *MSE* = 285.65, *p* = .04. More importantly, a significant difference was again found for the interference effect between sessions, *F*(1, 34) = 5.64, *MSE* = 382.4, *p* = .023, in which the interference effect was smaller after, as compared to before, the positive manipulation. These effects were not found in the neutral manipulation (Simon and interference effects: *F*(1,87) < 1, *F*(1,87) < 1).

**Fig 9:**
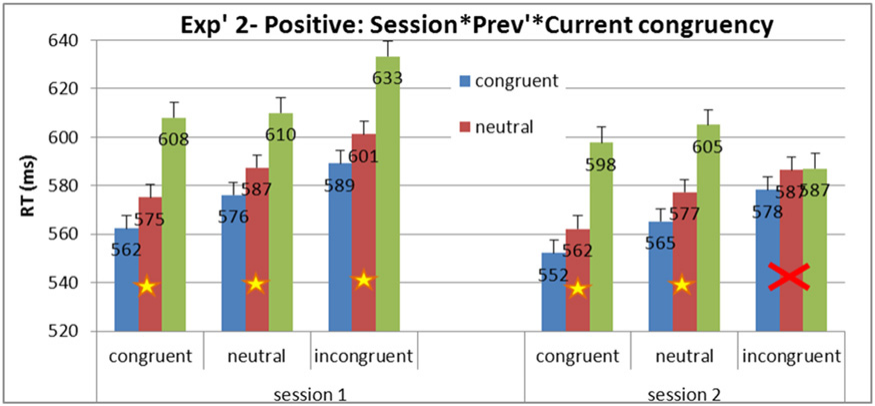
Experiment 2: Mean RTs and SE for congruency as a function of previous congruency between sessions in the positive manipulation. The maladaptive pattern of sequential analysis was present before the manipulation (Simon effect was significant after both congruent and incongruent trials), while an adaptive pattern of sequential analysis was present after the positive manipulation (Simon effect was significant after congruent, but not after incongruent, trials.

Once again, sequential analysis was carried out for the positive manipulation group (see Fig. 9). Planned comparisons of the Simon effect and interference effect were carried out between the ‘before manipulation’ session and the ‘after manipulation’ session. Replicating results of Experiment 1, a maladaptive Gratton was observed before the manipulation (the Simon effect was significant after both congruent and incongruent trials), *F*(1, 34) = 95.3, *MSE* = 196.87, *p* = .0001, and *F*(1, 34) = 54.9, *MSE* = 316.91, *p* = .0001, respectively. After the positive manipulation an adaptive Gratton pattern was observed (Simon effect significant after congruent but not after incongruent trials), *F*(1, 34) = 77.3, *MSE* = 241.3, *p* = .0001, and *F*(1, 34) = 2.5, *MSE* = 272.6, *p* = *ns*, respectively. The appearance of Gratton effect in the second session did not occur after the neutral manipulation, as the Simon effect was also significant after incongruent trials in the second session *F*(1, 34) = 90.59, *MSE* = 272.65, *p* = .0001 (see distribution plots for every participant in S4 Fig. for positive and S5 Fig. for neutral manipulations).

Regarding the duration of the manipulation effect, here again there was no difference between the blocks in the second session (decreased interference in both blocks of the second session *F*(1,34) < 1, and similar Gratton pattern, *F*(1,34) < 1)., strengthening the idea of long lasting effect of the positive manipulation in old age.

## Discussion

In the present study we examined whether social priming of cognitive states affects the inhibitory process in elderly adults by measuring the Simon effect, interference, and sequential analysis. For that purpose, participants were primed with the concept of a negative, neutral or positive cognitive state between two sessions of Simon task performance.

Results showed larger Simon and interference effects in elderly adults that were significantly decreased after the positive manipulation. This suggests a decrease in conflict interference as a result of positive cognitive state priming. Young adults were not influenced by the manipulations, in line with previous findings [52,35], suggesting that implicit priming affects those for whom the stereotype is self-relevant [53]. The Gratton pattern of sequential analyses that was present in young adults was not observed in elderly adults. Nevertheless, a healthy Gratton pattern appeared after the positive manipulation. Namely, the Simon effect was only present after congruent but not after incongruent trials. This implies that positive priming allowed elderly adults to benefit from solving one conflict after another.

The idea that thinking can influence behavior in a way that is outside of awareness is a striking, but not novel, idea [54]; (see also [55] for a review). In Smith and Queller’s [56] review of mental representations, semantic priming was discussed in the context of schema representations. Priming of a schema-relevant stimulus can activate a whole schema (e.g., ‘doctor’ priming can activate a whole schema of a hospital or medical environment). Higgins, Bargh, and Lombardi [57] proposed a *synapse metaphor*, according to which the activation of a schema is like a charge that decays with time. When one uses a schema it becomes fully “charged” with activation, which will then decay with time. Importantly, the synapse model suggests that frequency of use slows the decay of activation. This suggestion fits the aging self-stereotypes theory of Levy [38], and it is possible that the positive manipulation strengthens a positive age self-stereotype. This idea might have implications for considering rehabilitation interventions which make use of social priming.

Another way of understanding our results emerges from research related to the *resting state default mode network*. Since our results did not yield a significant difference between the blocks in the second session, it looks like the positive priming lasted longer and might have created a more constant change. Raichle et al. [58] suggested the existence of an organized, baseline default mode of brain function that is suspended during specific goal-directed behaviors. Damoiseaux et al. [59] demonstrated decreased activity in older versus younger participants in resting-state networks (RSN), containing the superior and middle frontal gyrus, posterior cingulate, middle temporal gyrus, and the superior parietal region. The authors found a correlation between reduced activity in RSN and less effective executive functioning/processing speed in the older group. It is plausible, then, that the positive manipulation in our experiment influenced the RSN by increasing certain activations and decreasing others, thereby allowing for improved executive functioning while performing the second session of the Simon task. This hypothesis needs to be explored in future research.

Because previous findings testing cognitive functions [36] showed that social priming effects last for only a few minutes, in contrast to our results, the duration of this influence must be further tested to lay the groundwork for social priming-based interventions.

In conclusion, our study supports the hypothesis that the implicit social concept of a positive cognitive condition in old age affects the inhibitory control process of the elderly and improves their cognitive abilities.

## Supporting Information

S1 Fig Experiment 1: Interference effect magnitudes for every participant in the senior group before and after each manipulation. (TIF)

S2 Fig Experiment 1: Interference effect magnitudes for every participant in the young adults group before and after each manipulation. (TIF)

S3 Fig Experiment 2: Interference effect magnitudes for every senior participant before and after each manipulation. (TIF)

S4 Fig Experiment 2: Positive manipulation—histogram of sequential effects for each showing Simon effect and interference effect for each participant after congruent and incongruent trials. (TIF)

S5 Fig Experiment 2: Neutral manipulation—histogram of sequential effects for each showing Simon effect and interference effect for each participant after congruent and incongruent trials. (TIF)

S1 File Supporting information: detailed description of a manipulation check, examining the influence of our manipulation on mood and motivation. (DOC)
