# Supplementary figures and images for: Social Priming Improves Cognitive Control in Elderly Adults—Evidence from the Simon Task
Source: PLoS One. 2015 Jan 30;10(1):e0117151. doi: 10.1371/journal.pone.0117151 (PMC4311990; doi:10.1371/journal.pone.0117151)

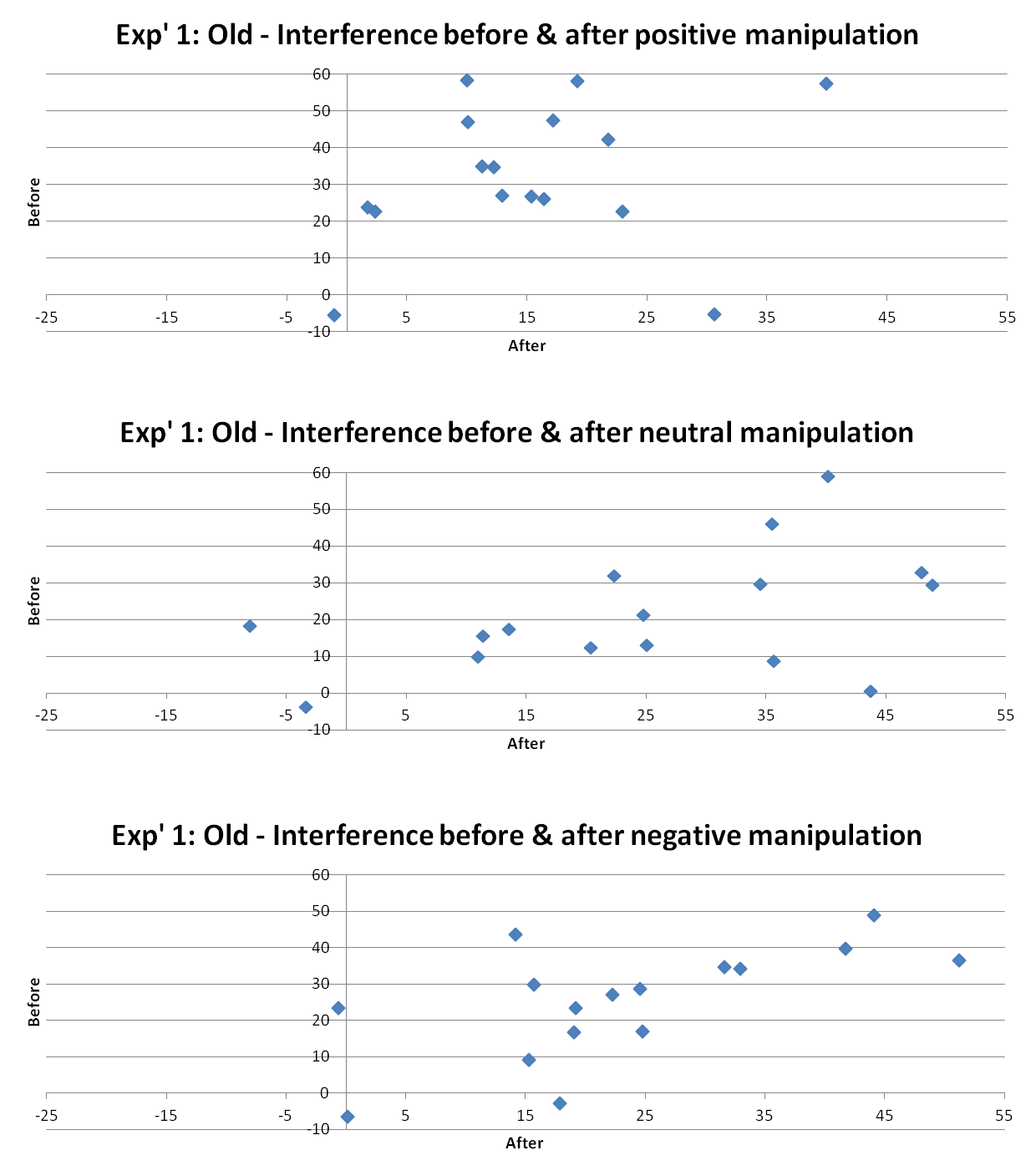

Supplement: S1 Fig — (TIF) [file pone.0117151.s001.tif]

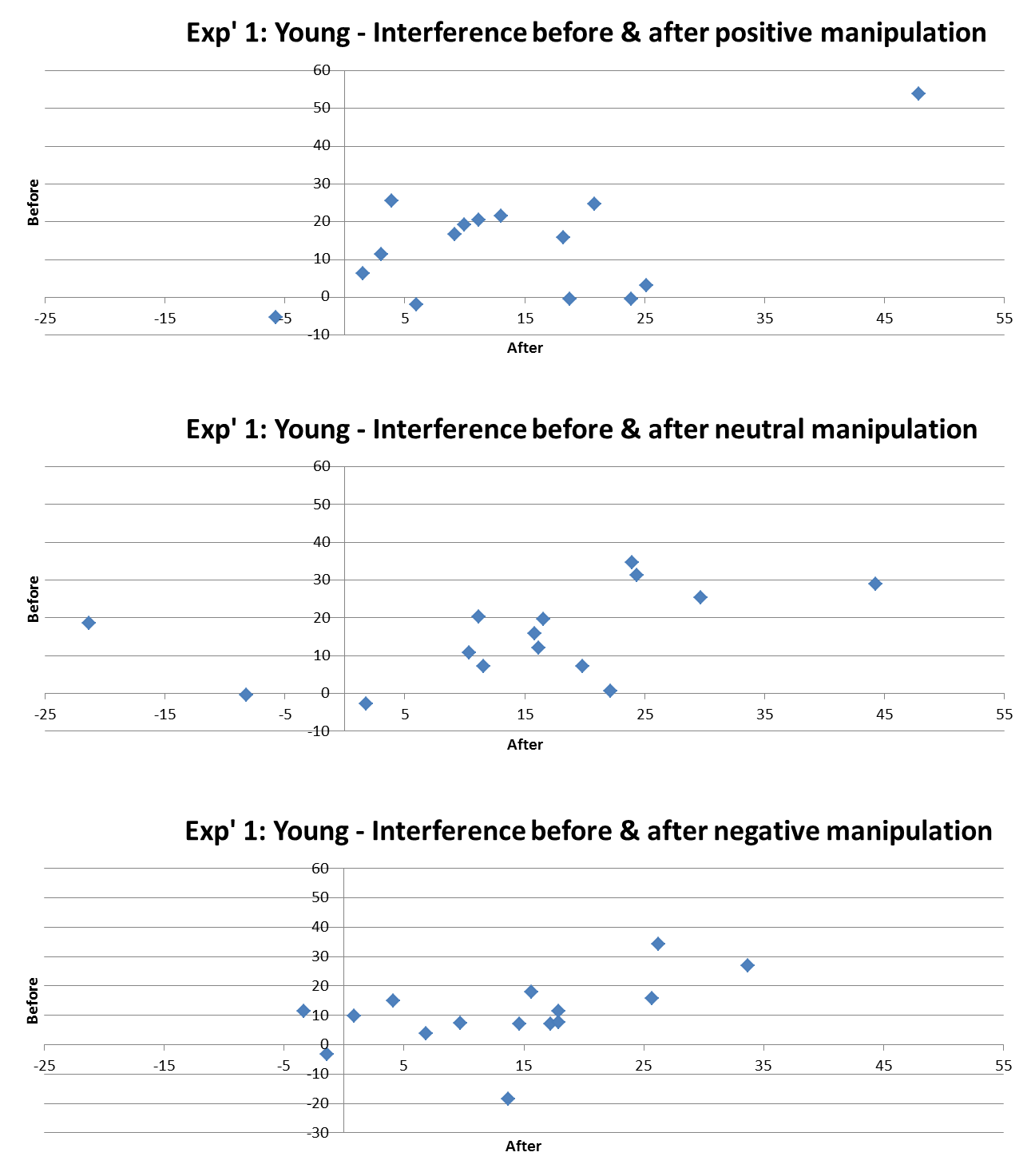

Supplement: S2 Fig — (TIF) [file pone.0117151.s002.TIF]

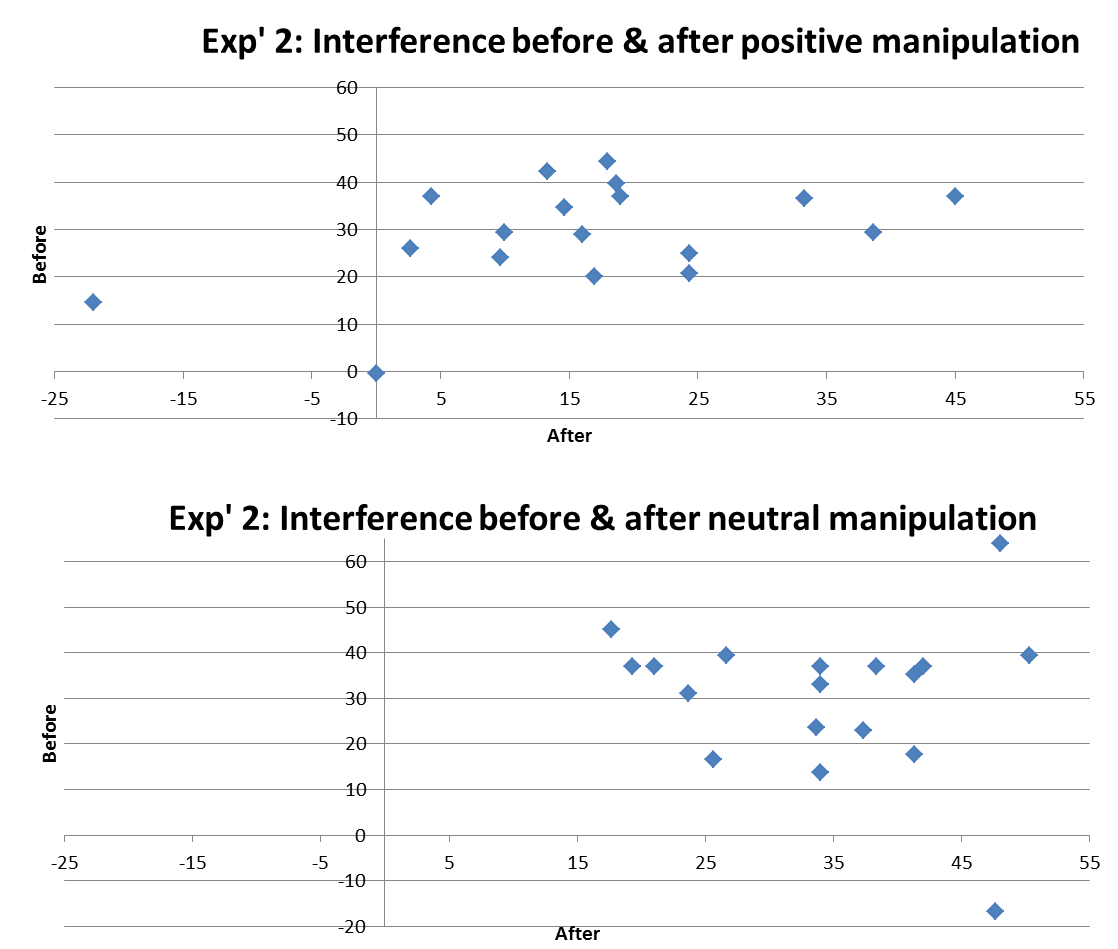

Supplement: S3 Fig — (TIF) [file pone.0117151.s003.TIF]

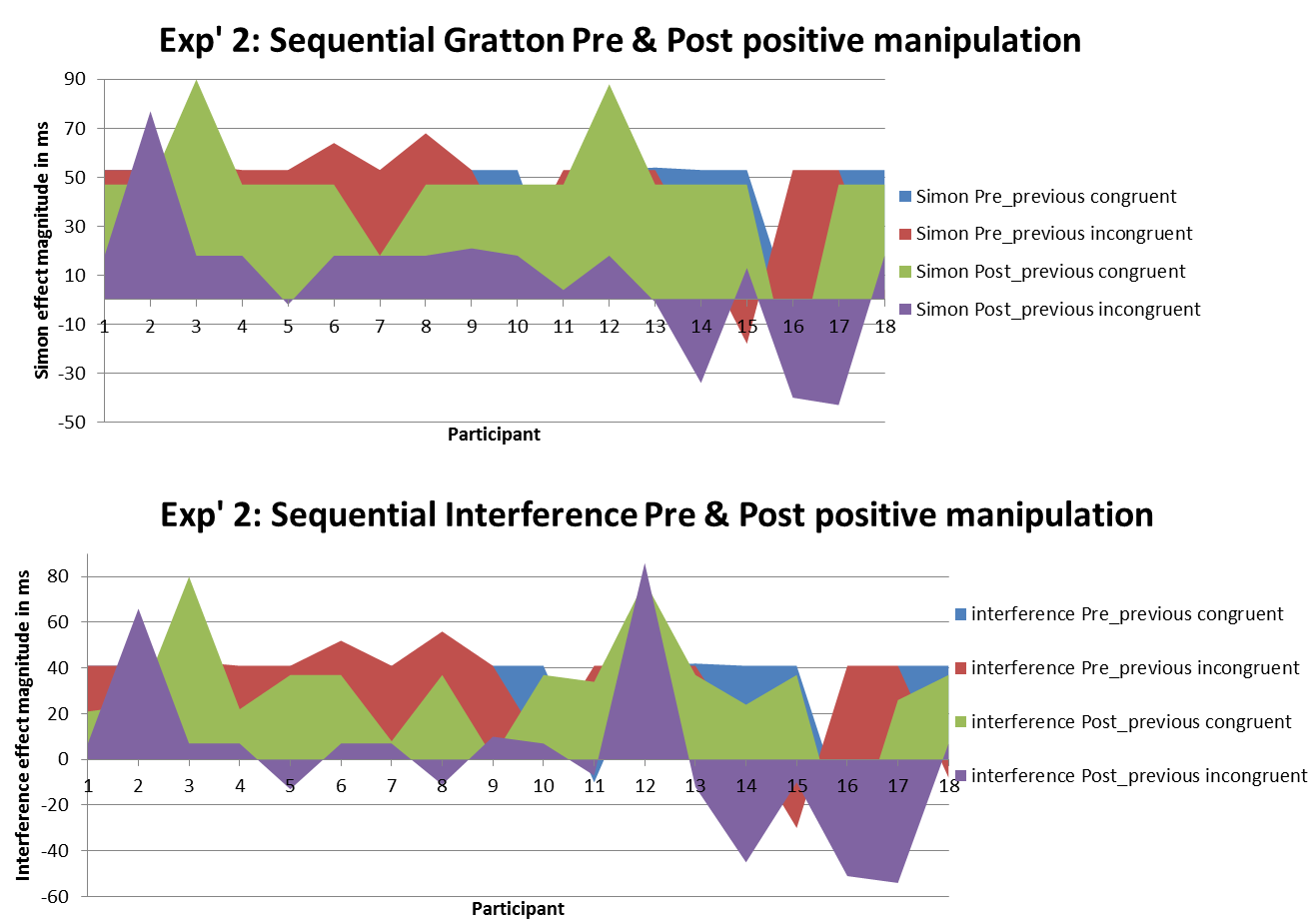

Supplement: S4 Fig — (TIF) [file pone.0117151.s004.TIF]

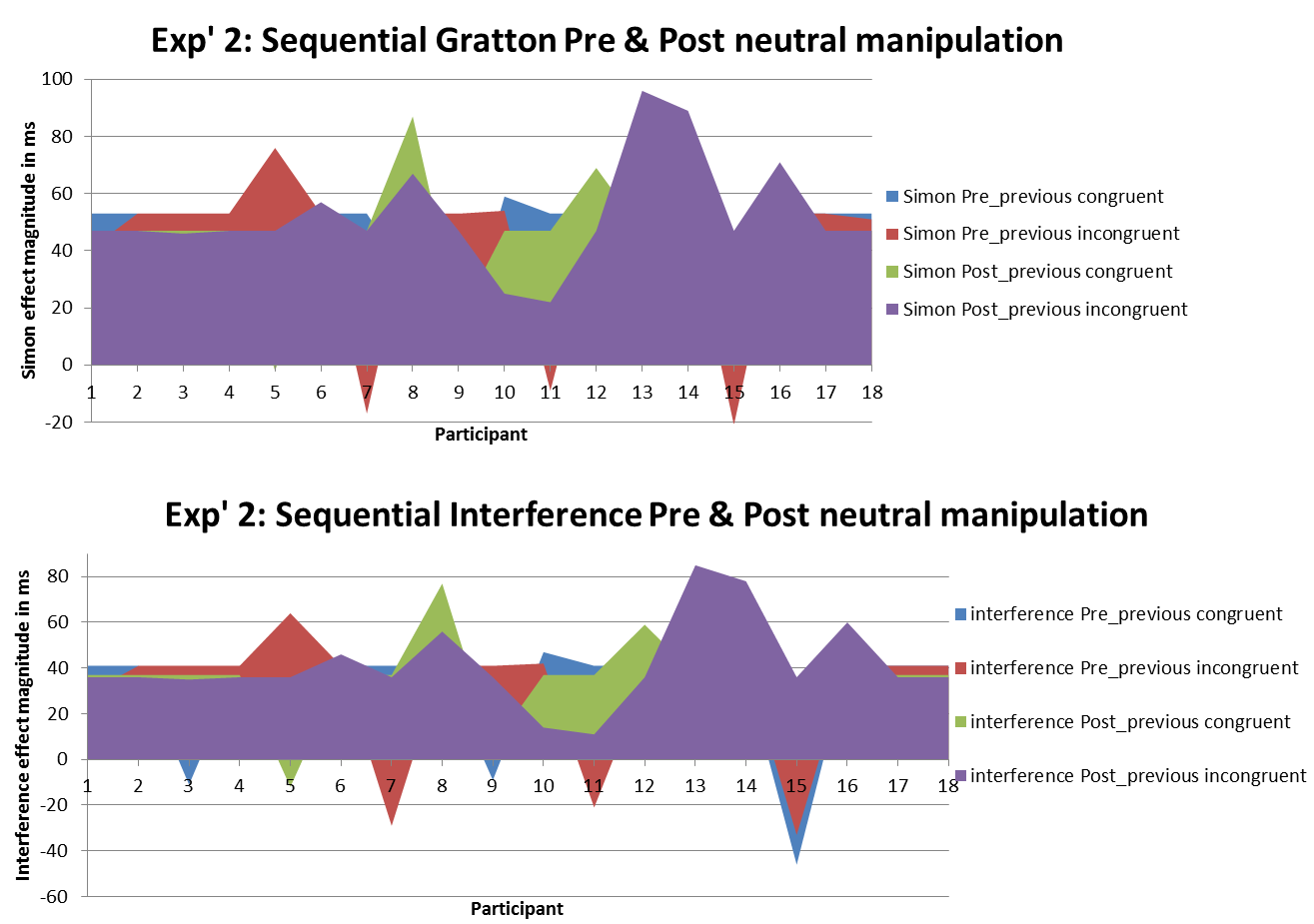

Supplement: S5 Fig — (TIF) [file pone.0117151.s005.TIF]
