# Supplementary material for: Social Priming Improves Cognitive Control in Elderly Adults—Evidence from the Simon Task
Source: PLoS One. 2015 Jan 30;10(1):e0117151. doi: 10.1371/journal.pone.0117151 (PMC4311990; doi:10.1371/journal.pone.0117151)
Supplement: S1 File — (DOC) [file pone.0117151.s006.doc]

***SI* - Supporting Information**

**Mood Inducing Examination – Manipulation Check**

A manipulation check experiment was conducted in order to rule out the possibility that the effect found (i.e., improved executive control performance in the experimental positive priming group) resulted from mood and motivation effects rather than from the actual cognitive social priming content.

**Materials and Method**

**Participants.** Twenty-four healthy elderly adults *(mean age* = 73.08 years, *SD* = 5.79) with normal or corrected-to-normal vision, without color blindness, participated in the experiment for payment. All were naïve regarding the purpose of the experiment. This study was conducted under a protocol approved by the Soroka Helsinki Ethics Committee.

**Mood questionnaire.** Mood was assessed via a 17-item mood visual analogue scale (VAS). Fourteen items were based on the PANAS-X (Watson, Clark, & Harkness, 1994) and three additional items were added to assess motivation (e.g., "I feel motivated"). For each mood item, participants rated their current feeling. The VAS was presented on a hard copy sheet and participants marked their response on a horizontal line, ranging from "not at all" (0) on the left side to "extremely" (100) on the right side. Cronbach’s alpha was .60 for the first mood rating, and .65 for the second mood rating.

**Procedure.** Participants completed mood ratings before and after they filled in the “social psychology” questionnaire. Two social priming manipulations that were identical to the main experiment were used: neutral (*n* = 12) and positive (*n* = 12).

**Results and Discussion**

Principal components factor analysis was applied to the ratings of the first mood assessment. Four independent factors representing dysphoric mood, motivated mood, relaxed mood, and anxious mood were identified. For each participant, we calculated a difference score (post-manipulation minus pre-manipulation) for each mood item and then averaged the items related to each factor. T-tests were used to examine whether the factors' difference score differed between the two priming groups. None of the t-tests yielded a significant effect: dysphoric mood: *t* (22) = -.33, *ns*; motivated mood: *t* (22) = -.37, *ns*; relaxed mood: *t* (22) = .45, *ns*; anxious mood: *t* (22) = .96, *ns*.

**Manipulation Check Conclusion**

The results of the manipulation check revealed that the positive and neutral priming did not change the mood or motivation induced in the participants. These results strengthen our claim that the effects found in the main experiment resulted from the specific priming content and not from a mood or motivation induction. Yet, In order to fully rule out the possibility that the priming manipulation affected mood or motivation an implicit measure of these aspects would be useful.

Reference

Watson, D., Clark, L. A., & Harkness, A. R. (1994). Structures of personality and their relevance to psychopathology. *Journal of Abnormal Psychology, 103*, 18.
